# Vitamin combination promotes ex vivo expansion of NK-92 cells by reprogramming glucose metabolism

**DOI:** 10.1186/s40643-022-00578-4

**Published:** 2022-08-26

**Authors:** Yan Fu, Yuying Chen, Zhepei Xie, Huimin Huang, Wen-Song Tan, Haibo Cai

**Affiliations:** State Key Laboratory of Bioreactor Engineering, East China University of Science and Technology, 130 Meilong Road, P. O. Box 309#, Shanghai, 200237 People’s Republic of China

**Keywords:** NK-92 cells, Ex vivo expansion, Response surface methodology, Vitamin concentration optimization, Glucose metabolism

## Abstract

Robust ex vivo expansion of NK-92 cells is essential for clinical immunotherapy. The vitamin B group is critical for the expansion and function of immune cells. This study optimized a vitamin combination by response surface methodology based on an in-house designed chemically defined serum-free medium EM. The serum-free medium EM-V4 with an optimal vitamin combination favoured ex vivo expansion of NK-92 cells. The characteristics of glucose metabolism of NK-92 cells in EM-V4 and the relationships between cell expansion and metabolism were investigated. NK-92 cells in EM-V4 underwent metabolic reprogramming. An elevated ratio of glucose-6-phosphate dehydrogenase/phosphofructokinase (G6PDH/PFK) indicated that NK-92 cells shifted towards the pentose phosphate pathway (PPP). An increase in the ratio of pyruvate dehydrogenase/lactate dehydrogenase (PDH/LDH) suggested that the cells shifted towards the Krebs (TCA) cycle, i.e., from glycolysis to aerobic metabolism. The enhanced ratio of oxygen consumption rate/extracellular acidification rate (OCR/ECAR) indicated that NK-92 cells were more reliant on mitochondrial respiration than on glycolysis. This shift provided more intermediate metabolites and energy for biosynthesis. Thus, EM-V4 accelerated biomass accumulation and energy production to promote NK-92 cell expansion by regulating the metabolic distribution. Our results provide valuable insight for the large-scale ex vivo expansion of clinically available NK-92 cells.

**Graphical Abstract:**

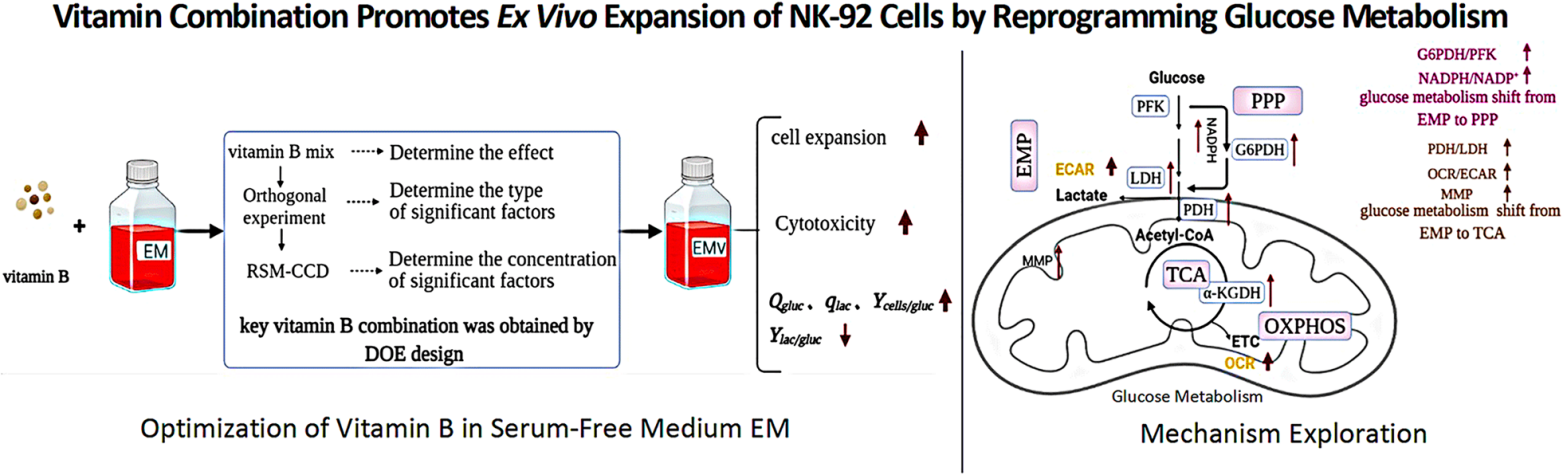

**Supplementary Information:**

The online version contains supplementary material available at 10.1186/s40643-022-00578-4.

## Introduction

Natural killer (NK) cells are effector lymphocytes important for antitumour and antiviral immune responses and act as potentially adaptive immune effectors. In addition, unlike T cells, NK cells do not cause graft-versus-host disease (Vivier et al. 2008; Waldhauer and Steinle 2008). However, only limited number of NK cells can be obtained initially, since these cells account for only 10% of lymphocytes. NK cells derived from the patients are often dysfunctional, and the cells derived from healthy donors usually require depletion of allogeneic T cells. In addition, clinically infused NK cells are always variable because of individual differences between the donors or patients (Cerwenka and Lanier 2016; Guillerey et al. 2016; Chiossone et al. 2018).

The interleukin (IL) 2-dependent NK-92 cell line (CD56^+^/CD3^−^) was isolated from non-Hodgkin’s lymphoma cells derived from a male patient (Gong et al. 1994; Hodge et al. 2002). NK-92 cells can be used to produce a large number of well-defined effector cell populations. NK-92 cells are “off the shelf therapeutic” for adoptive cancer immunotherapy based on natural killer cells and are readily available from a current (c)–GMP-compliant master cell bank; NK-92 cells have relatively stable growth characteristics and predictably higher cytotoxic activity than NK cells (Klingemann et al. 2016; Romanski et al. 2016; Suck et al. 2016). The established cell line expresses higher levels of perforin and granzyme B activity against a broad range of tumour cells. Accumulating experimental data have shown the clinical benefits with minimal side effects of NK-92 cells infused in patients with advanced cancer, such as haematological malignancies and lung cancer (Williams et al. 2015; Pockley et al. 2020). Tang et al. demonstrated that specific targeting by CAR–NK92 in acute myeloid leukaemia was characterized by good safety and high effectiveness (Tang et al. 2018). Although these cells have potential in clinical trials, specific protocols of NK-92 cell proliferation for clinical application have not been established (Tam et al. 2003; Chrobok et al. 2019). Large-scale ex vivo expansion of NK-92 cells under the conditions suitable for clinical practice remains challenging.

Major advances in immunology indicated that energy metabolism is important for the control of the function and growth of immune cells. Glucose is a substrate critical for the maintenance of cellular bioenergy, which is essential for cell proliferation (MacIver et al. 2008; Zhang et al. 2018; Jung et al. 2019). During glycolysis, glucose is broken down into two molecules of pyruvate that are transformed to lactate to quickly provide adenosine triphosphate (ATP) for cell expansion, which does not require oxygen. Oxidative phosphorylation (OXPHOS) is an oxygen-dependent process that can maximize the yield of ATP from glucose to maintain rapid biological processes. PPP (Pentose Phosphate Pathway) is an important nonoxidative arm of metabolism that generates key intermediates for nucleotide and fatty acid biosynthesis (Greiner et al. 1994; Han et al. 2016). Glycolysis and OXPHOS have been demonstrated to be essential for the development of NK cells. The regulation of metabolic distribution may be used to reversibly switch between quiescent and rapidly proliferating NK cells (Assmann et al. 2017; Poznanski et al. 2018). Lymphocyte metabolism can be regulated by the changes in the expression of metabolic genes and cell surface receptors and by feedback inhibition and other forms of allosteric regulation. In particular, enzyme regulation plays an especially prominent role in the regulation of metabolism by influencing the metabolic rates and fluxes to maintain metabolic homeostasis (Zhou et al. 2011; Pearce et al. 2013).

B vitamins are water-soluble vitamins involved in energy metabolism, methylation, DNA repair and immune regulation of the cells. This vitamin group often acts as coenzymes of the metabolic enzymes critical for mitochondrial and cellular functions (Depeint et al. 2006). Riboflavin is a precursor of flavin adenine dinucleotide and flavin mononucleotide (FMN), which are required for the flavoenzymes of the respiratory chain. Nicotinamide is central to the energy metabolism and is converted into nicotinamide adenine dinucleotide (NAD) and nicotinamide adenine dinucleotide phosphate (NADP), which participate in redox reactions in vivo (Schnellbaecher et al. 2019). In addition, inositol plays certain roles in cell expansion, acting as a precursor of phosphoinositides, membrane components, anchor molecules and signalling molecules in eukaryotic cells (Colazingari et al. 2014). These metabolites often form a tight network of interactions in the regulation of cellular metabolism. Moreover, the vitamin B group is important for cell proliferation and immune function. Riboflavin promotes ex vivo expansion of macrophages, and riboflavin deprivation has negative effects on macrophage activity and the immune response. Riboflavin-deficient mice had a higher risk of tumour development (Mazur-Bialy et al. 2015). Moreover, clinical data showed that riboflavin can be used as a therapeutic agent in disorders affecting mitochondrial energy metabolism (Bizukojc et al. 2007; Suwannasom et al. 2020). Nicotinamide was extensively studied. Clinical reports demonstrated that nicotinamide-dependent NK cells have higher toxicity and better expansion in patients with advanced multiple myeloma (MM) subjected to adoptive NK therapy. Nicotinamide supplementation can increase the proliferative capacity of mesenchymal stromal cells and umbilical cord blood-derived haematopoietic stem cells (Horwitz et al. 2014; Bachanova et al. 2019; Khorraminejad-Shirazi et al. 2020). Inositol was essential for blastocyst growth, which was crucial to the culture of preimplantation rabbit embryos in vitro (Inoue et al. 2002).

This study demonstrated that nicotinamide, riboflavin, and inositol were associated with amplification of NK-92 cells. The concentration of three substances was optimized by response surface methodology–central composite design (RSM–CCD) to promote ex vivo expansion of NK-92 cells. The glucose consumption rate, activities of key dehydrogenases and ATP production were investigated to better understand the effects of vitamin combinations on cell expansion. In general, this study can be used as a reference to achieve an extensive ex vivo expansion of NK-92 cells and therapeutic application.

## Materials and methods

### Ex vivo Expansion of NK-92 Cells

NK-92 cells were donated by the Bioengine Co., China. The cells were seeded at 1 × 10^5^ cells/ml in serum-free medium in the presence of 1,000 U/ml human recombinant IL-2 (Peprotech, USA). Cell cultures were maintained at 37 °C in a humidified incubator with 21% O_2_ and 5% CO_2_ in an atmosphere of nitrogen. NK-92 cells were counted every 2 days during the culture process, and the culture medium was sufficiently mixed before sampling. Then, fresh medium and IL-2 were added to maintain the cell density at 1 × 10^5^ cells/ml. The kinetics of NK-92 cell growth was calculated according to the following equations:

Expansion fold of total cells:

1 $$Y=\frac{{N}_{t}}{{N}_{0}}$$

where Y is the expansion fold of the cells, N_t_ is the number of the cells at indicated time t and N_0_ is the number of cells at time t_0_.

The specific growth rate:

2 $$\mu =\frac{ln{N}_{2}-ln{N}_{1}}{{t}_{2}-{t}_{1}}$$

where μ is the specific growth rate of NK-92 cells, N_1_ is the number of the cells at time t_1_ and N_2_ is the number of the cells at time t_2_.

### Experimental design and data analysis of optimization of vitamin concentrations

The concentrations of niacinamide (A) (Sigma-Aldrich, Germany, Catalog # N0636), riboflavin (B) (Sigma-Aldrich, Germany, Catalog # R9504) and inositol (C) (Sigma-Aldrich, Germany, Catalog # I7508) in the serum-free medium designed in the present study based on chemically defined EM were optimized for best cell expansion through 3-factor and 5-level CCD using Design Expert 8.0 software. Cell expansion after 8 days was selected as the response variable (Y). The concentration range of the factors was as follows: 16.50–40.00 µM niacinamide, 0.26–1.31 µM riboflavin and 26.97–238.75 µM inositol. An experimental design of 20 runs with 3 factors varying over 5 levels is summarized in Table 1. Optimum values of three variables were obtained after the response surface analysis.

**Table 1:** Central composite design and experimental data used for the response surface analysis

Runs	Factors			Total cell expansion, Y
	Nicotinamide(µM) A	Riboflavin(µM) B	Inositol(µM) C	
1	−1 (21.26)	−1 (0.48)	1 (195.82)	57.51
2	−1 (21.26)	−1 (0.48)	−1 (69.90)	79.80
3	1 (35.24)	1 (1.10)	−1 (69.90)	43.74
4	−1 (21.26)	1 (1.10)	1 (195.82)	63.04
5	1 (35.24)	1 (1.10)	1 (195.82)	56.83
6	0 (28.25)	0 (0.79)	0 (132.86)	94.57
7	0 (28.25)	0 (0.79)	0 (132.86)	83.48
8	−1 (21.26)	1 (1.10)	−1 (69.90)	44.48
9	0 (28.25)	0 (0.79)	0 (132.86)	99.52
10	1 (35.24)	−1 (0.48)	−1 (69.90)	76.27
11	0 (28.25)	0 (0.79)	0 (132.86)	89.47
12	1 (35.24)	−1 (0.48)	1 (195.82)	80.05
13	0 (28.25)	0 (0.79)	0 (132.86)	86.55
14	0 (28.25)	−1.69 (0.26)	0 (132.86)	64.49
15	1.69 (40.00)	0 (0.79)	0 (132.86)	72.04
16	−1.69 (16.50)	0 (0.79)	0 (132.86)	60.16
17	0 (28.25)	0 (0.79)	0 (132.86)	82.08
18	0 (28.25)	0 (0.79)	−1.69 (26.97)	72.39
19	0 (28.25)	1.69 (1.31)	0 (132.86)	55.05
20	0 (28.25)	0 (0.79)	1.69 (238.75)	54.22

### Immunophenotype analysis of expanded NK-92 cells

Approximately 7 × 10^5^ NK-92 cells were collected by centrifugation. After rinsing twice with antibody diluent, the cells were stained with PE-conjugated anti-human CD56 antibody (BD, USA, Catalog # 555,516) and FITC-conjugated anti-human CD3 antibody (BD, USA, Catalog # 555,332) for 30 min at 4 °C in the dark. Then, the cells were washed twice with antibody diluent and resuspended in 500 µl of antibody protection solution. Sample phenotypes were analysed by flow cytometry (BD, USA).

### Cytotoxicity assays of expanded NK-92 cells

Cytotoxicity of ex vivo expanded NK-92 cells was assessed by killing of K562 cells measured with cell counting kit-8 (CCK8) (Dojindo, Japan). NK-92 effector cells (E) target tumour K562 cells (T). Briefly, three experimental groups included a target cell group with (T), an effector cell group with (E) and an experimental group with cells at an E:T ratio of 5:1. The cells were suspended in 100 µl of the medium and seeded into 96-well microplates. After incubation for 4 h at 37 °C in an incubator, 10 µl CCK8 solution was added into each well, and the cells were incubated for another 2 h before detection of absorbance at 450 nm using a microplate reader. The cytotoxicity of NK-92 cells against K562 cells was calculated as follows:

3 $$cytotoxicity\, \%=\frac{{N}_{1}-({N}_{3}-{N}_{2})}{{N}_{1}}\times 100 \%$$

where N_1_, N_2_ and N_3_ represent the absorbance of the target cell group, effector cell group and experimental group, respectively.

The CD107a expression of cultured cells was assayed to evaluate cell degranulation. NK-92 cells were co-cultured with K562 cells at an E: T ratio of 5:1 for 4 h and then stained with mouse anti-human CD107a PE-Cy7-conjugated antibodies (BD, USA, Catalog # 561,348) for 30 min at 4 °C in dark. Besides, to carry out the intracellular granzyme B and perforin of NK-92 cells in cultures, cells were fixed, permeabilized and stained with antibodies of V450-granzyme B (BD, USA, Catalog # 563,389) and V450-perforin (BD, USA, Catalog # 563,393). The expression of CD107a, granzyme B and perforin were measured by flow cytometry.

### Detection and calculation of kinetic parameters

The culture supernatant was collected at specific timepoints by centrifugation to analyse the concentrations of glucose and lactic acid using a glucose assay kit and a lactate assay kit (Jiancheng Bioengineering Institute, China). The absorbance values were detected by a microplate reader. Relevant kinetic parameters were determined as follows:

Specific glucose consumption rate:

4 $${Q}_{gluc}=\frac{{C}_{1}-{C}_{2}}{{\int }_{{t}_{1}}^{{t}_{2}}Af(t)dt}$$

where C_1_ represents the concentration of glucose at time t_1_, C_2_ represents the concentration of glucose at time t_2_ and ${\int }_{{t}_{1}}^{{t}_{2}}Af\left(t\right)dt$ is the integral of the number of NK92 cells A from t_1_ to t_2_.

Specific lactic acid production rate:

5 $${q}_{lac}=\frac{{K}_{2}-{K}_{1}}{{\int }_{{t}_{1}}^{{t}_{2}}Af(t)dt}$$

where K_1_ represents the concentration of lactic acid at time t_1_, K_2_ represents the concentration of lactic acid at time t_2_ and ${\int }_{{t}_{1}}^{{t}_{2}}Af\left(t\right)dt$ is the integral of the number of NK-92 cells (A) from t_1_ to t_2_.

The yield coefficient of lactate to glucose:

6 $${Y}_{lac/gluc}=\frac{{q}_{lac}}{{Q}_{gluc}}$$

### Determination of activities of enzymes of glucose metabolism

The enzyme activities of phosphofructokinase (PFK), glucose-6-phosphate dehydrogenase (G6PDH), pyruvate dehydrogenase (PDH) and lactate dehydrogenase (LDH) in NK-92 cells were detected with PFK, G6PDH, PDH and LDH assay kits, respectively, following the manufacturer’s instructions (Comin Biotechnology, China).

### Extracellular flux assays

A Seahorse XFe 96 analyzer (Agilent Technologies, USA) was used to measure the OCR and ECAR of NK-92 cells cultured in various media. NK-92 cells (4 × 10^4^ per well) were seeded in Seahorse XF96 cell culture microplates, which were coated with polylysine overnight and washed twice using sterile water before plating the cells for the assay. The cells were cultured in the assay medium and incubated for an hour at 37 ℃ in an incubator without CO_2_. The glycolysis assay medium was glucose-free, and the mitochondrial assay medium contained 2 mM glutamine, 1 mM pyruvate and 10 mM glucose. For glycolytic stress tests, 10 mM glucose, 2 µM oligomycin and 30 mM 2-deoxyglucose were injected during the measurements. For the mitochondrial stress tests, 2 µM oligomycin, 0.5 µM FCCP and 2 µM rotenone/antimycin were sequentially added to the wells.

### Detection of intracellular metabolites

NK-92 cells were collected by centrifugation, and the intracellular metabolites ATP, NADP(H) and GSH were assayed using the corresponding assay kits, including an ATP assay kit, a NADP(H) assay kit and a GSH assay kit, respectively (Beyotime, China).

### Statistical analysis

The values are presented as the mean ± standard error. The significant differences were assessed by Student’s *t* test (two samples, one-tailed). P < 0.05 was considered statistically significant.

## Results

### Optimisation of concentrations of nicotinamide, riboflavin and inositol using CCD

RSM–CCD is an efficient mathematical and statistical method often used to optimize the experimental factors. The optimal concentrations of nicotinamide, riboflavin and inositol for the maximum fold expansion of NK-92 cells were determined using the 3-factor-5-level CCD method. The experimental design matrix and results are shown in Table 1. The corresponding second-order polynomial equation was as follows:

$${\text{Y}}\, = \,{88}.{85}\, + \,{2}.{\text{35A}} - {7}.{\text{43B}} - {1}.{\text{28C}} - {3}.{\text{24AB}}\, + \,{2}.{\text{57AC}}\, + \,{6}.{\text{27BC}} - {7}.{\text{88A}}^{{2}} - {1}0.{\text{12B}}^{{2}} - {8}.{\text{87C}}^{{2}} ,$$

where A, B and C are the concentrations of nicotinamide, riboflavin and inositol, respectively, and Y is the predicted total fold change in NK-92 cell expansion. Analysis of variance (ANOVA) indicated that the model *P* value is 0.0065, implying that the model is significant. The “lack of fit *P* value” of 0.1641 indicated that the effective mathematical equation is credible and lack of nonsignificant fitting is sufficient; thus, the equation can adequately reflect the actual relationships between the response variable and factors. In addition, the coefficient of determination (R^2^) was 0.86, indicating that the predicted and actual values were adequately fitted (Additional file 1: Table S1). All data indicated that the model prediction is credible.

**Fig. 1:**
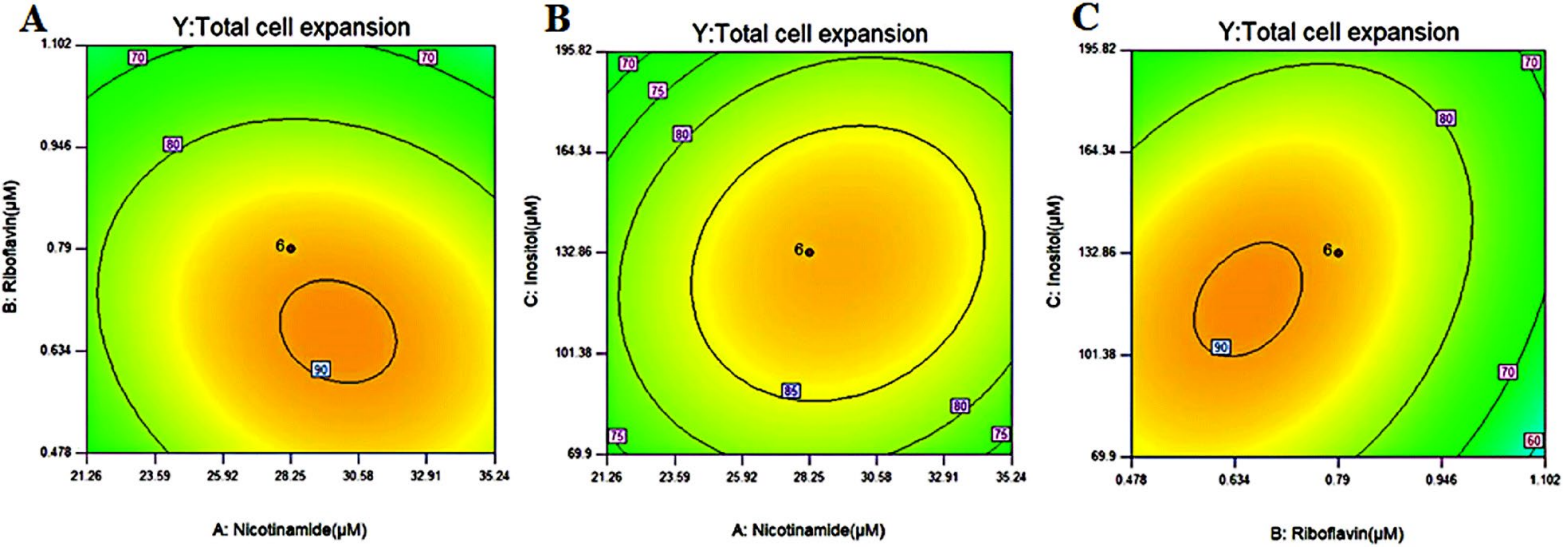
Contour plots for concentration optimization of nicotinamide, riboflavin and inositol: the effect of the mutual interactions of **A** nicotinamide and riboflavin, **B** nicotinamide and inositol, and **C** inositol and riboflavin on NK-92 cell expansion. (*n* = 3)

Intuitively, the 2D contour plots indicated the presence of a concentration point for each substance within the experimental concentration range optimally favourable for the best cell expansion (Fig. 1). The highest predicted value was obtained in the smallest ellipse in the contour diagram. Model prediction revealed that the maximum fold of cell expansion can be obtained when the optimal concentrations of A, B and C were 30.00 µM, 0.70 µM and 120.00 µM, respectively, and the predicted maximum response value was 91.00. Subsequently, the optimized EM-V4 medium was prepared to include this vitamin combination.

### Ex vivo expansion of NK-92 cells in EM-V4

**Fig. 2:**
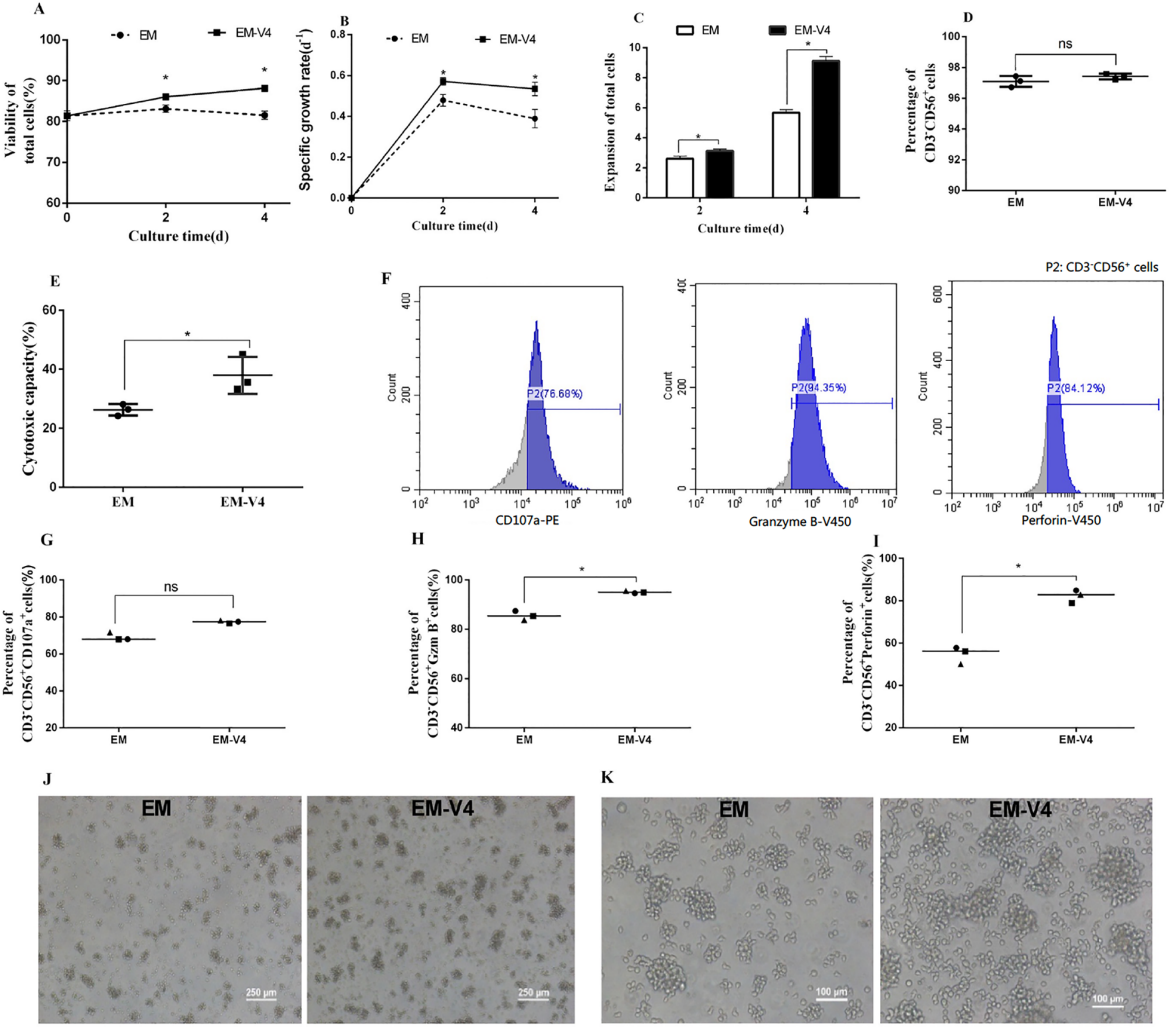
Ex vivo expansion characteristics of NK-92 cells. **A** Cell viability. **B** Specific growth rate of NK-92 cells. **C** Expansion fold of the cells. **D** Percentage of CD3^−^CD56^+^ cells. **E** Cytotoxicity of expanded NK-92 cells on day 4 at an E:T ratio of 5:1. **F** Representative flow cytometric analysis of CD107a^+^, granzyme B^+^ and perforin^+^ cells which grew in EM-V4; **G** Percentages of CD107a^+^ cells gated on CD3^−^CD56^+^ cells; **H** percentages of granzyme B^+^ cells gated on CD3^−^CD56^+^ cells; **I** percentages of perforin^+^ cells gated on CD3^−^CD56^+^ cells. **J** Light microscopic image of NK-92 cell morphology at 40 times magnification on day 4. **K** Light microscopic image of NK-92 cell morphology at 100 times magnification on day 4. (*P < 0.05, *n* = 3)

Cell expansion, phenotype and cytotoxicity were determined to verify the effect of EM-V4 on the growth of NK-92 cells. During the 4-day culture period, NK-92 cells in EM-V4 had higher viability than those in EM; correspondingly, the calculated indexes, μ and the fold expansion of total cells, were also higher (Fig. 2A–C). Meanwhile, cell expansion in longer period (8 days) was further analysed and the results also showed that cells in EM-V4 maintained higher viability, specific growth rate and expansion fold (Additional file 1: Fig. S1). Specifically, the fold expansion of total cells in EM-V4 was 110.9 ± 11.7 on day 8, which was significantly higher than the 42.2 ± 7.6 in EM. Obviously, EM-V4 improved the amplification rate of NK-92 cells. Moreover, the cell phenotype was analysed on day 4 by flow cytometry, and the percentages of CD3^−^CD56^+^ cells were comparable in both cultures (Fig. 2D). In addition, the data of Fig. 2E show that EM-V4-cultured NK-92 cells on day 4 had more robust cytotoxic ability based on an increase in the ability to kill K562 cells. In addition, higher percentage of CD107a^+^ cells, granzyme B^+^ cells and perforin^+^ cells in EM-V4 were shown in Fig. 2F–I, which could characterize the enhanced cytotoxicity well. Representative images of expanded NK-92 cells are shown in Fig. 2J, K. Brighter cells and higher number of the cell clusters were observed in EM-V4-cultured NK-92 cells, indicating a healthier cell proliferation status. Overall, these results demonstrated that EM-V4 favoured ex vivo expansion and stronger cytotoxicity of NK-92 cells without impairing the cell phenotype.

### Metabolic properties of NK-92 cells expanded in EM-V4

**Fig. 3:**
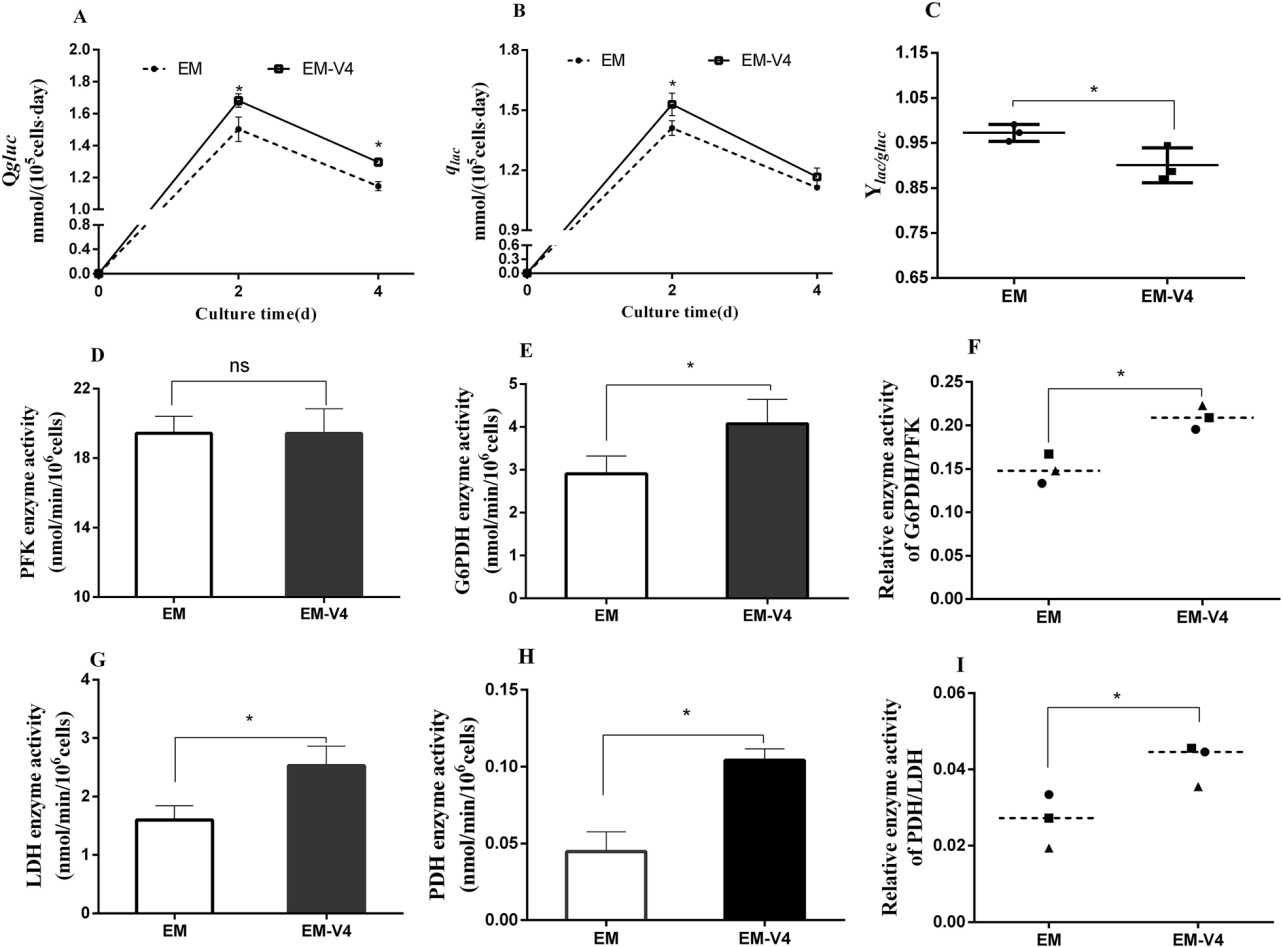
Characteristics of glucose metabolism of NK92 cells. **A** Specific glucose consumption rates of the cells (*Q*_*gluc*_). **B** Specific lactate production rates of the cells (*q*_*lac*_). **C** Yield coefficient of lactate to glucose conversion (Y_*lac/gluc*_). **D** PFK activity. **E** G6PDH activity. **F** Relative G6PDH/PFK activity. **G** LDH activity. **H** PDH activity. **I** Relative PDH/LDH activity. (*P < 0.05, *n* = 3)

Glucose metabolism was investigated to determine how EM-V4 influenced the ex vivo proliferation of NK-92 cells. Specifically, kinetic parameters in both cultures were analysed. The glucose and lactate concentrations in both culture supernatants were measured every 2 days, and the results are shown in Additional file 1: Fig. S2, which revealed that NK-92 cells in EM-V4 had higher glucose consumption and lactic acid production. Subsequently, *Q*_*gluc*_ was calculated using Eq. (4) to analyse the rate of glucose consumption. As shown in Fig. 3A, the *Q*_*gluc*_ of the cells in the two cultures exhibited a similar trend of an increase on day 2 compared to that on day 4, indicating that the glucose consumption rate of the cells in the two cultures initially increased and subsequently decreased. Furthermore, higher *Q*_*gluc*_ of NK-92 cells indicated that the cells cultured in EM-V4 had a higher glucose metabolism rate. The *q*_*lac*_ of the cells displayed a trend similar to that of *Q*_*gluc*_. However, the differences in a decrease in *q*_*lac*_ were detected on day 4 in the two cultures (Fig. 3B). Considering the lower Y_*lac/gluc*_ of the cells in EM-V4, EM-V4 reduced the coefficient of lactate to glucose conversion of the cells and improved the TCA cycle distribution ratio between glycolysis and aerobic metabolism (Fig. 3C).

Furthermore, the key enzymes of the glucose metabolism were assayed to determine the mechanisms by which EM-V4 regulates glucose metabolism. Glucose enters the cells and is directly involved in glycolysis and PPP, which are regulated by the rate-limiting enzymes PFK and G6PDH, respectively. Then, pyruvate is generated in the cytoplasm. On the one hand, pyruvate can be used in the PDH-catalysed reaction to produce acetyl-CoA that enters the TCA cycle; on the other hand, pyruvate can be used in the LDH-catalysed reaction to produce lactic acid. Two metabolic distribution ratios were considered in the present study. In the case of glycolysis and PPP, NK-92 cells in both cultures displayed comparable PFK activities; however, EM-V4-cultured cells had significantly higher G6PDH activity than that in EM-cultured cells (Fig. 3D, E), indicating that EM-V4 improved glucose consumption of the cells by increasing the PPP metabolic flux. A higher G6PDH/PFK value suggested that the cells shifted to PPP (Fig. 3F). Moreover, LDH and PDH activities of NK-92 cells were investigated. The results showed that EM-V4 upregulated cellular LDH and PDH activities, especially the PDH activity (Fig. 3G, H). The improved PDH/LDH value of EM-V4-cultured NK-92 cells indicated that the TCA distribution ratio was increased (Fig. 3I). Thus, EM-V4 increased the rate of glucose metabolism and governed metabolic reprogramming by regulating the activities of key dehydrogenases of NK-92 cells. Improved PPP metabolism ratio and TCA distribution percentage may enhance the generation of the substrates and energy for cell proliferation.

**Fig. 4:**
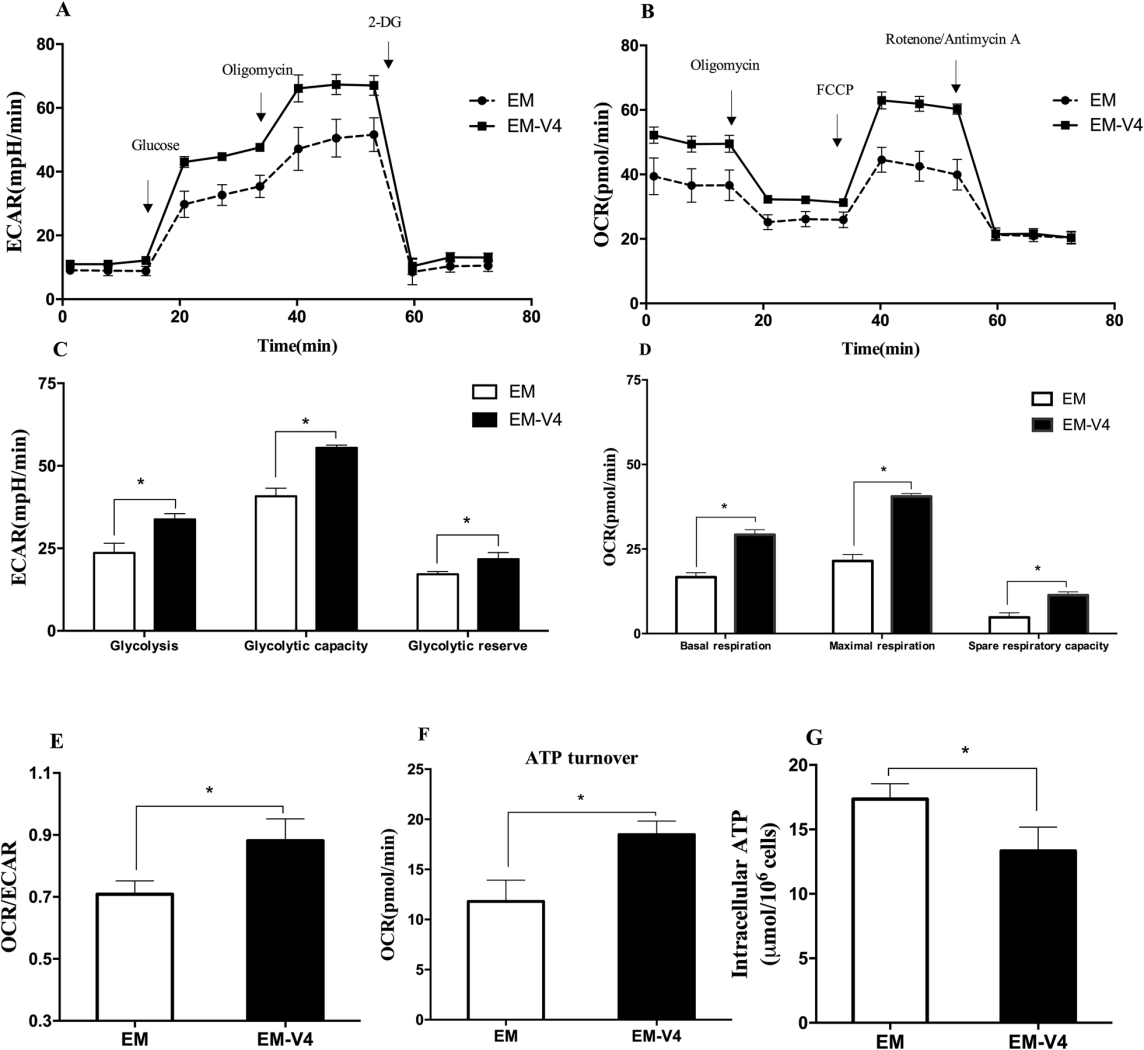
Extracellular acidification rate (ECAR) and oxygen consumption rate (OCR) of NK-92 cells. **A** ECAR of the glycolytic rate. **B** OCR of mitochondrial oxygen consumption rate. **C** Basal ECAR, glycolytic capacity and glycolytic reserve of the cells. **D** Basal OCR, maximal respiration and spare respiration capacity of the cells. **E** OCR/ECAR ratio. **F** ATP turnover. **G** Intracellular ATP level. (*P < 0.05, *n* = 3)

Similarly, the ECAR (an indicator of glycolysis) and OCR (an indicator of OXPHOS) values were determined using a Seahorse analyser. Figure 4A–D shows that EM-V4-activated NK-92 cells had increased ECAR and OCR, indicating that EM-V4 improved the energy metabolism of the cells. The higher OCR/ECAR ratio suggested that EM-V4 promoted OXPHOS more than glycolysis in NK-92 cells (Fig. 4E). Furthermore, the increased ATP production capacity in the mitochondria was detected (Fig. 4F). ATP is the key energy donor crucial for cellular processes in lymphocytes, including both cell proliferation and immune response. The intracellular ATP content of NK-92 cells was measured. Interestingly, slightly lower ATP levels were observed in EM-V4-stimulated NK-92 cells on day 4 (Fig. 4G). Considering that intracellular ATP levels are a result of the consumption and production, large amounts of ATP may be expended for biosynthesis. Hence, these results demonstrated that NK-92 cells in EM-V4 had enhanced ATP synthesis and relied more on OXPHOS to produce ATP for faster cell replication.

**Fig. 5:**
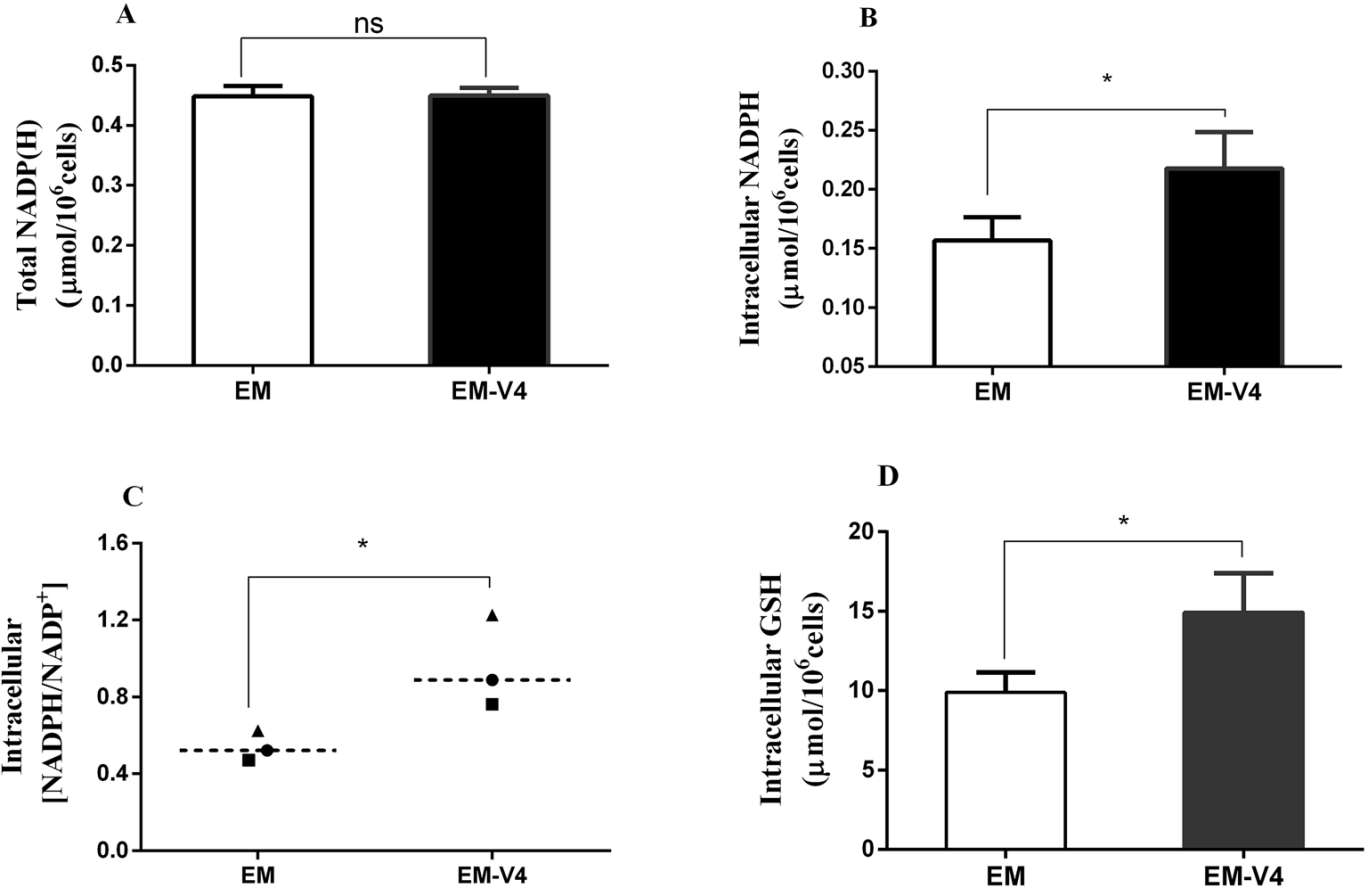
Content of intracellular metabolites is related to the physiological state of NK-92 cells. **A** Total NADP(H). **B** NADPH. **C** NADPH/NADP.^+^ ratio. **D** Intracellular GSH level. (*P < 0.05, *n* = 3)

Moreover, intracellular NADP(H) and GSH were determined to investigate the physiological state of the cells. No significant differences were observed in the total NADP(H) levels in the two cultures (Fig. 5A); however, EM-V4-cultured NK-92 cells had higher NADPH levels and NADPH/NADP^+^ ratio (Fig. 5B, C). In addition, elevated GSH content was detected in EM-V4-cultured NK-92 cells (Fig. 5D). Thus, increased NADPH and GSH levels maintained a higher cellular redox state, which favoured cell expansion in vitro.

## Discussion

Adoptive NK cell therapy is a promising strategy against cancer. Although comprehensive characterisation of metabolic pathways during NK cell development has not been reported, immature NK cells are highly reliant on glucose metabolism than other lymphocytes. NK cell activation is mainly fuelled by glucose. In addition, activated NK cells undergo metabolic reprogramming, which is significant for cell proliferation and functional response (Gardiner 2019). The vitamin B family is involved in glucose metabolism, influencing enzyme activity, and has a profound impact on the regulation of metabolic pathways. Some vitamins can effectively promote the growth and function of various lymphocytes (Grudzien & Rapak 2018). Thus, niacinamide, riboflavin and inositol were considered in the present study, and an optimized vitamin combination was obtained by CCD (Fig. 1). Our data demonstrated that the optimal EM-V4 medium, including the optimized vitamin combination, improved the expansion and cytotoxicity of NK-92 cells (Fig. 2).

Efficient glucose metabolism has been demonstrated to be critically important for NK cell responses. Hence, kinetic parameters during the culture process and key enzyme activities of glucose metabolism were analysed. The results showed that the optimal vitamin combination improved the glucose metabolism rate and regulated metabolic flux by regulating the key metabolic enzymes (Fig. 3). Specifically, the vitamin combination upregulated the G6PDH, LDH and PDH activities and maintained the PFK activity. We hypothesised that nicotinamide and riboflavin may act as coenzymes regulating dehydrogenase activity through their active forms. Nicotinamide can transform into NAD^+^ and elevate deacetylase activity to improve glucose metabolism and extend the longevity of worms (Mouchiroud et al. 2013). The optimal vitamin combination apparently upregulated glucose metabolism and induced changes in the distribution of metabolites by regulating the enzyme activities to promote cell expansion.

Accelerated glucose consumption of the cells may result in higher energy production. Our results revealed that NK-92 cells in EM-V4 produced higher amount of energy (Fig. 4). The higher ECAR and OCR values of the cells indicated that EM-V4 improved ATP synthesis by enhancing both glycolysis and OXPHOS. In addition, the higher OCR/ECAR ratio indicated that EM-V4 had a greater impact on OXPHOS of the cells than that on glycolysis, which was consistent with an increase in the PDH/LDH ratio and a decrease in *Y*_*lac/gluc*_. Demonstrated shift from glycolysis to mitochondrial metabolism of NK-92 cells may favour the maximal level of ATP derived from glucose for faster biomass accumulation (Zhang et al. 2019). Activated NK cells are characterized by a shift in relative ATP reliance from OXPHOS to glycolysis, which may be a reaction to increased energy demands within a short time period; however, mature NK cells still mainly rely on OXPHOS to support a change in energy needs (O’Brien and Finlay 2019). Moreover, nicotinamide can promote the proliferation of human primary keratinocytes by elevating OXPHOS (Tan et al. 2019). Theoretically, nicotinamide and riboflavin may be used as the substrates and electron acceptors to participate in the respiratory chain to promote OXPHOS. In addition, inositol included in EM-V4 may promote fatty acid metabolism together with other B vitamins to regulate mitochondrial respiration (Burton and Wells 1976). Therefore, the vitamin combination may induce metabolic reprogramming and enhance cellular ATP synthesis to improve cell proliferation.

Healthy cell physiology provides better support of cell growth. It has been demonstrated that nicotinamide can upregulate the expression of antioxidant reductase and delay ageing in mice (Zhang et al. 2016). In addition, riboflavin can elevate the expression of antioxidant proteins in HepG2 cells (Xin et al. 2017). Higher intracellular NADPH levels and NADPH/NADP^+^ values of EM-V4-cultured cells detected in the present study were consistent with an increase in the metabolic flux of PPP. In addition, NADPH can induce the synthesis of GSH. Therefore, an increase in the cellular GSH content may result from an increase in the NADPH level (Fig. 5). An increase in the contents of these antioxidants may maintain a more active state of the cells.

**Fig. 6:**
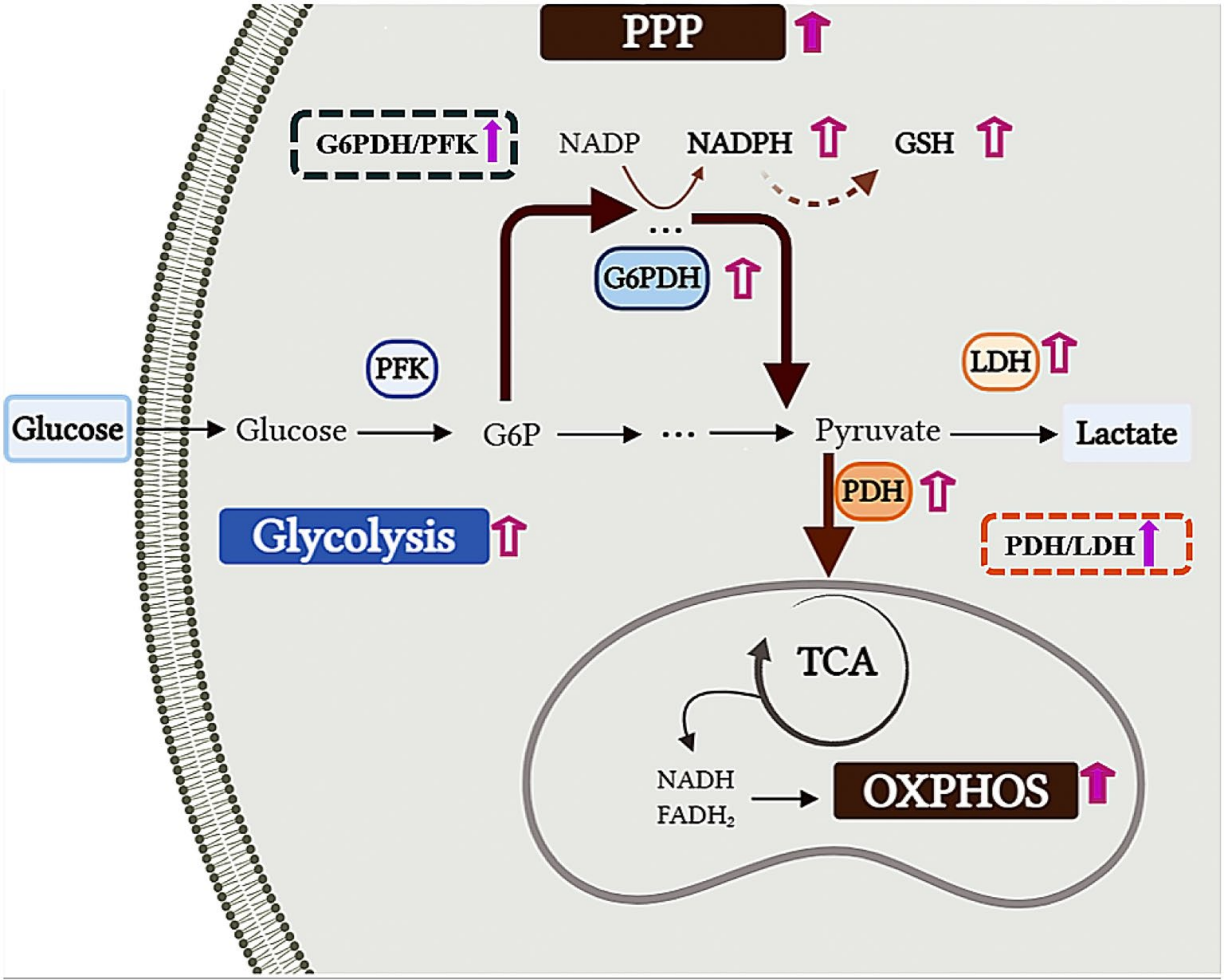
Overview of glucose metabolism distribution in EM-V4-cultured NK92 cells. Upregulated metabolic pathways, enzyme activities and intracellular metabolites are indicated with red arrows

Thus, the vitamin combination upregulated the activities of the key dehydrogenases of NK-92 cells to accelerate the glucose consumption rate and promote metabolic migration. Enhanced PPP flux provided the higher amount of the biomaterials for biomass accumulation, and migration from glycolysis to aerobic metabolism supplied sufficient energy for better ex vivo expansion of NK-92 cells (Fig. 6).

## Conclusions

In this study, we optimized a vitamin combination, which could favor ex vivo expansion of NK-92 cells. In addition, the effect of vitamin combination on cell expansion was investigated. Results demonstrated that the optimal vitamin combination could induce glucose metabolism reprogramming and enhance energy metabolism by upregulating key dehydrogenase activities of NK-92 cells. Besides, it maintained a better cellular redox state. That will lay a solid foundation for scale-up ex vivo expansion of NK-92 cells and serum-free medium development to provide a valuable guidance for therapeutic application.Additional file: As per journal requirements, every additional file must have a corresponding caption. In this regard, please be informed that the caption of Additional file [1] was taken from the additional e-file itself. Please advise if action taken appropriate and amend if necessary.We regarded it as appropriate action.

### Supplementary Information

**Additional file 1:Table S1. **ANOVA for response surface quadratic model. **Fig. S1. Ex vivo** expansion of NK-92 cells for 8 days. (A) Cell viability. (B) Specific growth rate of NK-92 cells. (C) Expansion fold of the cells. (*P < 0.05, n = 3). **Fig. S2.** Time-profiles of glucose consumption and lactate production of NK-92 cells in two kinds of mediums. (A) Glucose concentration. (B) Lactate concentration. (n = 3).

## Data Availability

All data generated or analyzed during this study are included in this article and its supplementary information files.

## Abbreviations

NK: Natural Killer
G6PDH: Glucose-6-phosphate dehydrogenase
PFK: Phosphofructokinase
PPP: Pentose phosphate pathway
PDH: Pyruvate dehydrogenase
LDH: Lactate dehydrogenase
TCA: Tricarboxylic acid
OCR: Oxygen consumption rate
ECAR: Extracellular acidification rate
IL: Interleukin
GMP: Good manufacturing practice
CAR: Chimeric antigen receptor
ATP: Adenosine triphosphate
OXPHOS: Oxidative phosphorylation
FMN: Flavin mononucleotide
NAD: Nicotinamide adenine dinucleotide
NADP: Nicotinamide adenine dinucleotide phosphate
MM: Multiple myeloma
GSH: Glutathione
RSM–CCD: Response surface methodology–central composite design
CCK8: Cell counting kit-8
ANOVA: Analysis of variance
2D: 2-Dimensional
CoA: Coenzyme A
