# Supplementary material for: Vitamin combination promotes ex vivo expansion of NK-92 cells by reprogramming glucose metabolism
Source: Bioresour Bioprocess. 2022 Aug 26;9(1):87. doi: 10.1186/s40643-022-00578-4 (PMC10991583; doi:10.1186/s40643-022-00578-4)
Supplement: Supplementary file 1 — Additional file 1:Table S1. ANOVA for response surface quadratic model. Fig. S1. Ex vivo expansion of NK-92 cells for 8 days. (A) Cell viability. (B) Specific growth rate of NK-92 cells. (C) Expansion fold of the cells. (*P < 0.05, n = 3). Fig. S2. Time-profiles of glucose consumption and lactate production of NK-92 cells in two kinds of mediums. (A) Glucose concentration. (B) Lactate concentration. (n = 3). [file 40643_2022_578_MOESM1_ESM.docx]

**Additional file 1 Files**

**Additional file 1: Table S1.** ANOVA for response surface quadratic model.

| Source | DF | SS | MS | F | P |
| --- | --- | --- | --- | --- | --- |
| Model | 9 | 4357.23 | 470.31 | 6.06 | 0.0065* |
| A | 1 | 75.18 | 75.18 | 0.97 | 0.3506 |
| B | 1 | 753.24 | 753.24 | 9.71 | 0.0124* |
| C | 1 | 22.22 | 22.22 | 0.29 | 0.6055 |
| AB | 1 | 84.24 | 84.24 | 1.09 | 0.3245 |
| AC | 1 | 53.03 | 53.03 | 0.68 | 0.4297 |
| BC | 1 | 314.6 | 314.60 | 4.06 | 0.0749 |
| A^2^ | 1 | 895.27 | 895.27 | 11.54 | 0.0079* |
| B^2^ | 1 | 1475.35 | 1475.35 | 19.02 | 0.0018* |
| C^2^ | 1 | 1133.68 | 1133.68 | 14.62 | 0.0041* |
| Lack of Fit | 5 | 546.13 | 109.23 | 2.87 | 0.17 |
| Cor Total | 19 | 5008.66 |  |  |  |


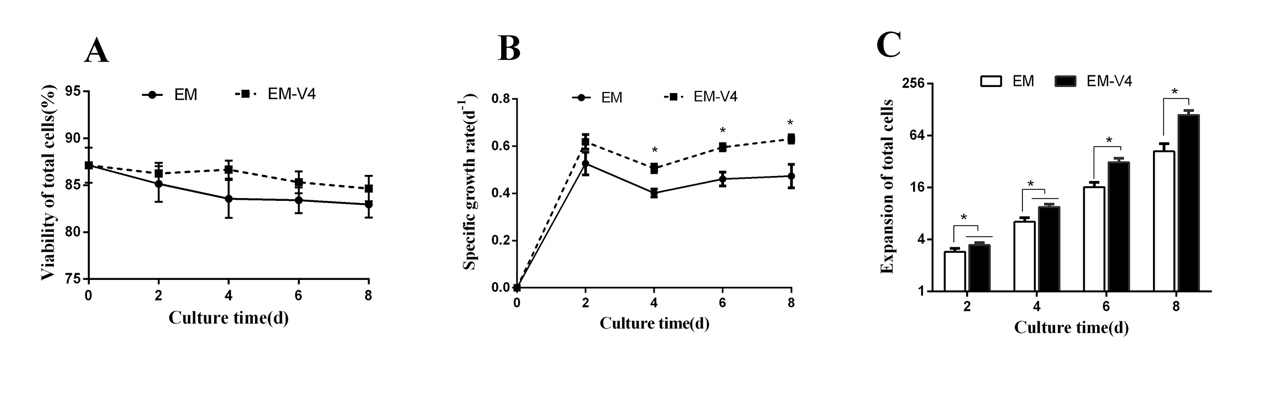


**Additional file 2: Fig. S1.** *Ex vivo* expansion of NK-92 cells for 8 days. (A) Cell viability. (B) Specific growth rate of NK-92 cells. (C) Expansion fold of the cells. (*P < 0.05, n = 3).


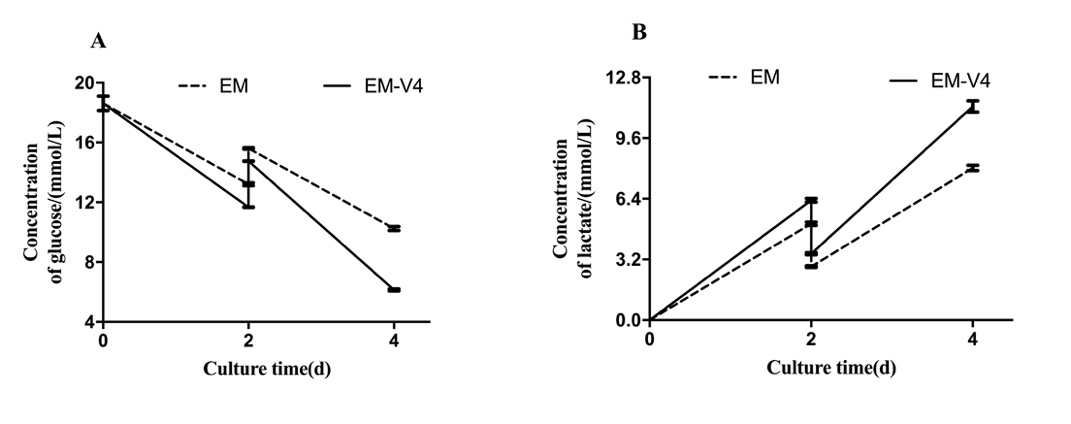


**Additional file 3: Fig. S2.** Time-profiles of glucose consumption and lactate production of NK-92 cells in two kinds of mediums. (A) Glucose concentration. (B) Lactate concentration. (n = 3).
